# Goals and Self-Efficacy Beliefs During the Initial COVID-19 Lockdown: A Mixed Methods Analysis

**DOI:** 10.3389/fpsyg.2020.559114

**Published:** 2021-01-12

**Authors:** Laura Ritchie, Daniel Cervone, Benjamin T. Sharpe

**Affiliations:** ^1^University of Chichester Conservatoire, University of Chichester, Chichester, United Kingdom; ^2^Department of Psychology, University of Illinois at Chicago, Chicago, IL, United States; ^3^Institute of Sport, University of Chichester, Chichester, United Kingdom

**Keywords:** goals, self-efficacy, COVID-19, coping, social psychology, lockdown, projects

## Abstract

This study aimed to capture how the coronavirus disease 2019 (COVID-19) crisis disrupted and affected individuals’ goal pursuits and self-efficacy beliefs early during the lockdown phase of COVID-19. Participants impacted by lockdown regulations accessed an online questionnaire during a 10-day window from the end of March to early April 2020 and reported a significant personal goal toward which they had been working, and then completed quantitative and qualitative survey items tapping self-efficacy beliefs for goal achievement, subjective caring about the goal during disrupted world events, and current pursuit or abandonment of the goal. The findings from both quantitative and qualitative measures demonstrated a significant drop in self-efficacy beliefs from before to during the pandemic with a large effect based on whether people thought they could still achieve their goal under current conditions. Over two-thirds of the sample was unsure or did not believe they could still carry out their goal, and over a quarter either abandoned or were uncertain they could pursue the goal. Despite this, people continued to care about their goals. Reasons for abandonment and strategies for coping with goals within the lockdown and beyond are discussed.

## Introduction

Goals give meaning to life. People experience greater well-being and a higher sense of fulfillment when their days include activities structured by, and directed toward, personally significant aims (Sheldon and Elliot, 1999; Emmons, 2003). Evidence from both personality/social and clinical science attests to this (e.g., Ho et al., 2010). Even individuals experiencing severe psychopathology anticipate personal well-being when envisioning a future in which they attain self-nominated personal goals (Coughlan et al., 2017).

Many significant life goals have a quality that is well-captured by the concept of “projects” (Little, 2007). Personal projects are interrelated sets of activities organized toward an overall aim (e.g., “prepare to apply to medical school,” “find a new job so I can quit this current one”). Projects not only organize everyday actions but also foster a coherent sense of self; self-concept is reflected in and developed by the pursuit of valued personal projects (Bruner, 1990; Little, 1993).

When people commit themselves to a meaningful project, they usually pursue it over a substantial period of time. For example, Langan-Fox (1991) assessed university students’ personal goals at two time periods 5 months apart. As compared with an older-adult population, one might expect that such younger adults would experience instability of goals as they consider alternate personal and professional futures. Yet, among both female and male students, goal content was highly stable. Projects persist partly as a result of becoming elements of enduring “life stories,” that is, narratively structured conceptions of one’s life path and personal identity (McAdams, 1996). Empirically, the themes contained in personal goals and life stories are strongly related (McGregor et al., 2006).

Little (2011) emphasizes that goals are not just mental contents stored in the head. Personal strivings are fundamentally intertwined with the social contexts in which one lives. People often can sustain their pursuit of a personal goal in “the felicitous case” in which they work toward “projects that are meaningful, manageable, and supported by the eco-setting” (Little, 2011, p. 80). But what happens in the infelicitous case, when the eco-setting withdraws its support?

One answer to this question may be found in the study of goal appraisals, that is, people ongoing evaluations of their goal-directed activity. When establishing and working toward goals, people engage in strategic evaluation of their progress, using goal appraisals and aspects of self-regulation (Bembenutty et al., 2013). One major appraisal is coping capability, or appraisals of self-efficacy, (Bandura, 1977, 1986), and the unexpected disruption presented by the coronavirus disease 2019 (COVID-19) pandemic provides a particular set of globally felt conditions in which to consider people’s understanding of their capabilities, through self-efficacy, and adherence to goals. The relation between self-efficacy appraisals and goal commitments can vary from one context to another, even in ordinary times. Often, self-efficacy contributes to goal setting; people are less likely to pursue goals when they doubt their capability for success (Locke and Latham, 2006; Bardach et al., 2020). However, in some contexts, people persist on goal-directed activities even in the absence of high-efficacy expectations. This occurs, for example, when goal achievement is critical to avoiding substantial personal loss (Shah and Higgins, 1997; Senko and Freund, 2015; for a meta-analytic review, see Huang, 2016) or when the goal-directed activity is an expression of personal values and the intuitive, “integrated” self (Kuhl et al., 2015).

Basic research in personality, social, and developmental psychology establishes the causal impact of self-efficacy and goal processes on social behavior and well-being. For example, in longitudinal research, self-efficacy predicts psychosocial outcomes even after accounting for the role of personality traits (Caprara et al., 2004), goal setting predicts achievement and interest in activities (Scherrer et al., 2020), and personal goals impact subjective well-being (Brunstein, 1993) which, in turn, is found to facilitate re-engagement with meaningful life goals (Haase et al., 2020). With this basic research as our background, in the present study, we sought to portray the nature of goal pursuit and self-efficacy beliefs at a uniquely disruptive moment in recent world history, namely, the early period of the social “lockdown” necessitated by the rapid spread of COVID-19 in the early months of the year.

The worldwide disruption created by the COVID-19 pandemic has fundamentally changed people’s lives physically and psychologically (Jiang, 2020; Qiu et al., 2020; Remuzzi and Remuzzi, 2020), with several studies outlining the negative psychological impact that forced quarantine can have on the population (Brooks et al., 2020), citing reductions in positive emotions, sleep disturbances, and increased feelings of anger and anxiety (Cava et al., 2005; Roy et al., 2020). The reduction in social contact and steps taken to deal with the psychological impact of this (Bzdok and Dunbar, 2020) has added a potentially significant disruption to the populations’ previous goal pursuits. Goals may now yield to new challenges such as limited access to food, financial worries, sudden need for employment, the care of isolated family members, or reductions to health (Cipolletta and Ortu, 2020; Fraenkel and Cho, 2020; Wilms et al., 2020). In addition to its vast biomedical and economic costs, there likely was a psychological cost associated with the disruption of valued personal projects by imposed social and mobility restrictions. What was the nature and magnitude of that goal disruption? How did people cope with their altered life circumstances? These are questions addressed in the present report.

## Materials and Methods

### The Present Research

We conducted a mixed-methods survey of goals, self-efficacy beliefs, and potential goal disruptions in the early period of the lockdown in the COVID-19 crisis. Three aspects of the survey are of note. The first is its conceptual basis, which was a social-cognitive orientation (Bandura, 1986), in which self-reflection on one’s capabilities, the setting of goals, and self-regulatory efforts are central to emotion, motivation, and achievement (also see Sarrazin et al., 1996; Caprara et al., 2011; Zimmerman et al., 2017). Survey items focused on three classes of thoughts and feelings about self-identified projects that are consistent with this perspective: (1) self-efficacy beliefs for goal achievement (Bandura, 1997); (2) subjective caring about the goal in light of disrupted world events (a variable associated with self-evaluative reactions to those that are central to social-cognitive analyses of self-regulation; Bandura and Cervone, 1983; Cervone et al., 1991); and (3) commitment to goals, that is, whether people saw themselves as still pursuing the projects that, prior to the pandemic, had been central to their everyday lives.

A second feature is the type of survey items we included; our use of both quantitative and qualitative measures is unique within the self-efficacy literature, which has almost exclusively relied on quantitative self-ratings of people’s self-efficacy appraisals. Given the utter novelty of the COVID-19 outbreak, we judged the inclusion of open-ended qualitative measures necessary for learning about people’s beliefs and experience in the midst of this pandemic. Self-efficacy researchers have continued to develop and validate questionnaires to better capture the construct within a specific domain (Bandura, 2006; Bong, 2006; Ritchie and Williamon, 2011; Axboe et al., 2016); however, the need for a qualitative approach that moves beyond the traditional questionnaire has been suggested (Ritchie, in press), but no studies to date have investigated the possible comparability of qualitative and quantitative methods.

Allowing participants to speak, in their own terms, about their goals, experiences, and coping strategies enabled us to explore both the phenomenology of engagement, motivation, and processes of personal agency while navigating iterative and unforeseen challenges toward achievement. Given the mixed-methods data source, we report quantitative analyses, human-based coding of narrative text, and computational natural language processing of syntax and sentiment in that text.

The third aspect of the survey was practical. Early in a pandemic, people have a lot to do other than filling out surveys. We focused our survey exclusively on the set of variables (described above) of maximal interest, in an effort to maintain clarity while eliciting full free-text responses that captured a sense of the person, their outlook, and their investment in the goal. We also deliberately constrained the time frame of survey administration to relatively narrow time window early in the COVID-19 lockdown period when lifestyle changes were newly imposed and still unfolding. Previously, “normal” life was still within recent experience, allowing a snapshot of comparative outlooks. Had we allowed the data collection period to extend, there was a risk that people would begin to develop a catalog of adaptive behaviors in light of these new conditions. Our goal was to gain insight into people’s efforts to sustain their projects at the onset of this dramatically challenging moment in history.

### Participants

The participants (*n* = 161) were aged 19–80 years, *M* = 45.70 (SD = 14.86), and lived in the United Kingdom (*n* = 101), the United States (*n* = 31), and 11 other countries (*n* = 23) across five continents. Six individuals chose not to declare their location. On an 11-point bipolar measure of gender identity, 56 participants identified themselves as strongly male (1–3), 94 strongly female (9–11), nine identified with the middle (4–8), and two individuals did not identify on this scale. Participants were notified of the study *via* online academic and social media networks and voluntarily completed a “Pivotal Moments and Goals” (PMG) questionnaire, administered *via* Qualtrics^1^, between March 27th and April 6th, 2020. This 10-day window allowed a snapshot of views early in the COVID-19 pandemic—a period of rapid social change in which people comprehended the significance of the crisis and governments instituted lockdowns (the British Prime Minister doing so on March 23rd) (Government United Kingdom, 2020) and travel bans (the United States Centers for Disease Control and Prevention issued a strong travel advisory for New York, Connecticut, and New Jersey on March 28th) (CDC, 2020).

### Materials

#### Goal Description

After a consent statement which noted the study’s ethical approval (from the University of Chichester, Approval No. 1920-25), the PMG acknowledged a (consensually recognized) pivotal moment caused by global events and stated our interest in how the social changes triggered by this event may have impacted people’s personal goals. Participants were asked to contemplate an important project they had prioritized prior to the event and to describe it in a provided text box.

#### Retrospective Self-Efficacy Beliefs

Participants next rated their confidence that they could do the goal *prior to* COVID-19 events on a 1–100 sliding scale (Bandura, 1977). As a qualitative measure of prior self-efficacy beliefs, participants were asked to describe this confidence in words in two to three sentences. Responses to this and subsequent open-ended items were typed into on-screen text boxes.

The quantitative self-efficacy questionnaire adheres to common practice in the self-efficacy literature, in which self-efficacy beliefs (perceived capabilities to carry out courses of action and achieve aims) are assessed on 100-point scales and without provision of the construct name in questionnaires (Bandura, 2006; Bong, 2006). Qualitative self-efficacy reports are employed rarely (Williams, 1990), yet follow naturally from the fact that self-reflective thinking is primarily formulated through the tools of natural language (Cervone and Lott, 2007). The free prose responses gathered here supplement numerical ratings, allowing a richer, self-guided assessment of self-efficacy and step outside the limitations of traditional empirical questionnaires.

#### Contemporaneous Self-Efficacy Beliefs

Current self-efficacy beliefs for goal pursuit were assessed in three steps: a multiple-choice item asking if “you can do this now” (yes, no, unsure); a 1–100 scale rating of confidence that you “can still do this”; and a two to three sentence description of this current confidence.

Before answering subsequent questions, participants were instructed to pause to consider their ideas before responding.

#### Caring

Participants were next asked to indicate whether they still care about the goal (yes, no, unsure) and to describe “how and why you care” in two to three sentences.

#### Contemporaneous Goal Pursuit

Participants were asked in a multiple-choice format (yes, no, genuinely undecided) whether they were still pursuing their stated goals. They next completed an open-ended report based on this multiple-choice response in which they were asked to indicate either (1) if no, why no; (2) if yes, if anything has changed in goal pursuit; or (3) if uncertain, “Can you say something about this?”

Demographic information (age, gender identity, and country of residence) was collected last so as not to distract people from essential questions. Importantly, all respondents understood that data would be published *via* open source repositories; thus, responses to demographic questions were optional, allowing respondents to preserve as much privacy as they wished.

### Exploratory Data Analysis Methods

All freely written responses were analyzed with AWS Amazon Comprehend^2^ to confirm English as the language used and then analyze the syntax and its sentiment (positive, neutral, negative, and mixed) (Ribeiro et al., 2016). After an initial review of text-based responses, it came to light that 10 cases incorrectly completed the questionnaire, and these were removed from the sample. These participants either used negatively framed responses which produced negatively coded sentiment analysis (e.g., “I had no reason to think I couldn’t do it unless I went under a bus”) or misunderstood the directive of the questions (e.g., failed to choose a goal from before world events unfolded).

The sentiment analysis uses logistic regression to assign probability that the text sentient is positive, neutral, negative, or mixed. For example, the first two text questions describe confidence to carry out goals; therefore, negative sentiment scores reflect the probability the text describes this confidence negatively. Thus, 1 minus the negative score provides a number representing total probability showing the text sentiment is not negative (e.g., 1 minus a negative value of 0.2 becomes 0.8). These numbers were calculated and correlated with the corresponding self-efficacy scores to ensure the text reflected the participant’s numerical confidence rating.

Goals and the reasons for pursuing or abandoning goals were qualitatively coded separately by two researchers.

## Results

### Goals, Self-Efficacy, Caring, and Goal Pursuit

#### Goal Description

For descriptive purposes, we first classified the content of participants’ self-described goals into categories that were derived rationally by the investigators subsequent to the reading of all goal content. Goals could be classified into one of six categories, with varying observed frequency of response: educational (*n* = 39, 24.22%), professional (*n* = 36, 22.36%), change place of residence (*n* = 12, 7.45%), house repair (*n* = 6, 3.73%), travel (*n* = 38, 23.60%), and a range of projects involving personal development (*n* = 30, 18.63%).

#### Retrospective and Contemporaneous Self-Efficacy Beliefs

Retrospective and contemporaneous self-efficacy beliefs differed markedly. On the 100-point strength of the self-efficacy rating scale, participants reported high pre-COVID-19 self-efficacy scores, *M* = 84.6 (SD = 20.8, SE = 1.64), but much lower contemporaneous, post-outbreak self-efficacy, *M* = 45.6 (SD = 34.7, SE = 2.73). Retrospective and contemporaneous strength of self-efficacy differed highly significantly, *t*(160) = 11.6, *p* < 0.001.

**FIGURE 1:**
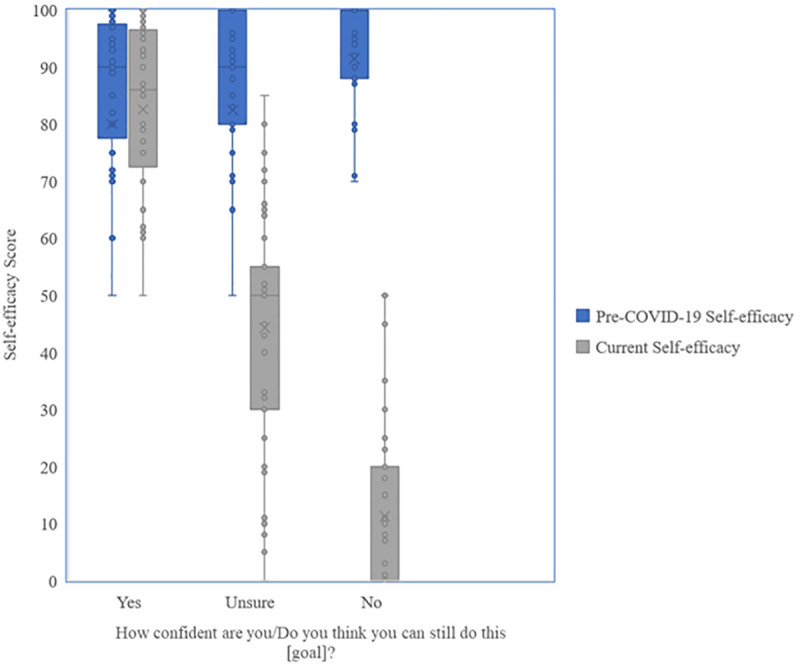
Comparison of mean pre-COVID-19 and current self-efficacy scores in response to the question “Can you do this (goal)?”

Complementary results resulted from the analysis of the responses to the multiple-choice item asking, “Can you still do this [goal] now?” On this item, 31.68% of people responded yes, 35.40% unsure, and 32.92% no. These three subgroups of participants differed highly significantly in their 100-point scale rating self-efficacy beliefs, as would be expected, *F*(2, 158) = 53.6, *p* < 0.001, and η_p_^2^ = 0.404 (Cohen, 1988). See Figure 1 for the change in mean pre-COVID-19 and current self-efficacy scores.

Strong correlations were demonstrated between the pre-COVID-19 self-efficacy scores and verbally reported confidence sentiment scores (*r* = 0.376, *p* < 0.001) and between the current self-efficacy and corresponding verbal confidence sentiment scores (*r* = 0.415, *p* < 0.001), demonstrating these verbal descriptions to be a representative measure of self-efficacy.

Results of a one-way repeated-measures ANOVA using the verbal confidence sentiment scores from the text describing current self-efficacy mirrored results achieved with the current numerical self-efficacy measure, with a highly significant result showing a very large effect with significant differences between groups [*F*(2, 158) = 14.0, *p* < 0.001, η_p_^2^ = 0.151].

#### Caring About the Goal

In contrast to the considerable variability in pre- and contemporaneous self-efficacy beliefs, there was relative uniformity in caring about the goal. On the measure of caring, 89.44% of people reported yes that they still cared, 4.35% were unsure, and 6.21% no longer cared about their goal.

#### Contemporaneous Goal Pursuit

A major question was whether people were continuing to pursue their goal despite the restrictions imposed as a result of COVID-19. Notwithstanding the significance of the goals and high levels of caring, 43 participants (26.70%) indicated that they either were no longer pursuing their goal (*n* = 24) or were undecided about whether they could pursue it (*n* = 19). Of the 118 reporting continued pursuit of their goal, 116 confirmed they still did care and only two reported uncertainty about caring. These two had extenuating personal circumstances that rendered the goal doable but no longer of value. For example, one person reporting uncertainty about caring was planning a course that was canceled, yet they still intended to carry out the planning.

Qualitative coding of textual goal responses revealed reasons both why participants abandoned or maintained the pursuit of their goals. (For the purposes of simplifying this descriptive analysis of textual responses, we combined into a single category the participants who were not pursuing and were undecided about whether they were still pursuing their goal). Those who were no longer actively pursuing their goals (*n* = 43) cited external, often physical factors (*n* = 18, 41.86%); general uncertainty and difficulty in making long-term plans (*n* = 12, 27.91%); and a shift in priorities where they had to readjust due to caring or health responsibilities (*n* = 11, 25.58%). Two responses were unique in their reasons and did not categorize.

Those who maintained their goals (*n* = 118), despite the increased uncertainty because of lockdown and drop in self-efficacy beliefs, presented a range of perspectives and strategies. Some saw COVID-19 as not changing or impacting their goal (*n* = 27, 22.88%), whereas a larger group demonstrated either problem-focused coping (*n* = 47, 39.83%) or emotion-focused coping (*n* = 18, 15.25%) (Folkman and Lazarus, 1980). Others stated they intended to continue with the goal but exhibited a “holding pattern” (*n* = 16, 13.56%), essentially hitting pause during COVID-19. Two of these “holding” people also demonstrated aspects of problem- and emotion-focused coping strategies, for example:

“Yes, by monitoring the situation and wait for news about when we can start planning trips again so we can go. Obviously it won’t be at the same time as my partner’s birthday but at least we will go away and do what we had in mind.”

In order to better understand the qualitative responses of those who continued pursuing their goals, we established a rationally based taxonomy to analyze the text responses based on a syntax analysis. Four groups of strategic behaviors, as shown in Table 1, demonstrated active pursuit, engagement, and adaption in relation to goal pursuit in response to the altered conditions imposed by lockdown.

**TABLE 1:** Strategic behaviors reported by those continuing to pursue their goals.

**Planning**	**Engaging with others**	**Enhanced personal awareness/engagement**	**Strategic thinking**
For the completion of tasks	Accessing assistance with goals	Engaging in dedicated physical activity	Adapting to a new pattern of activity (at home)
For the modification of events/goals	Phoning friends	Self-care—sleep, nutrition	Navigating challenges of new media (online)
For “being ready” to do when restrictions allow (e.g., social distancing or restrictions of movement are not imposed)	Speaking with experts/professionals	New hobbies/activities	New ways to reorient the goal
Maintaining a schedule	Trading ideas	Increased focus and attention	Adaptation and implementation of new methods to circumvent the lockdown challenge
Control of personal time (daily schedules) and engagement with activity because of being at home as a result of lockdown	Collaborative working	Increased contentment, pleasure in tasks	Greater attention to detail
For the future	Using new media to maintain contact (zoom)		Dedicated, analytical thinking about methods, direction, and timing of tasks
	Developing external facing materials (eBooks, websites)	Internal motivation	Active monitoring of personal progress and events
		More personal involvement and a sense of agency	Internal reflection
			

### Relations Between Self-Efficacy Beliefs and Goal Pursuits

Our cross-sectional design of course does not allow an analysis of potential causal relations between self-efficacy beliefs and goal pursuits. We analyzed the relation between these variables for descriptive purposes, as a way of characterizing patterns of thinking about personal projects that people experienced during the early lockdown period of COVID-19.

#### Goal Pursuits and Self-Efficacy Beliefs

There is more than one way to describe the relations among goal pursuits and self-efficacy beliefs. One is to examine self-efficacy beliefs among three groups of participants, namely, those who were undecided about whether they were still pursuing their goal, were no longer pursuing their goal, and were pursuing their goal (i.e., the subgroups of participants who responded in these ways to the multiple-choice format question about contemporaneous goal pursuit). For these three groups, we analyzed two self-efficacy variables: (1) the magnitude of change (generally a decline) in strength of self-efficacy from pre- to during the COVID-19 lockdown and (2) AWS sentiment analysis scores obtained by coding the verbal responses from the free-response contemporaneous self-efficacy item. Both variables were standardized for ease of presentation of results.

**FIGURE 2:**
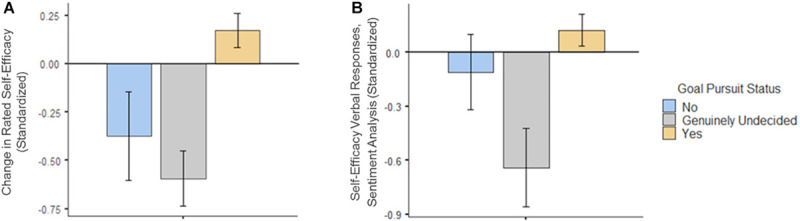
Variations in quantitative self-efficacy ratings **(A)** and sentiment analysis of verbalized self-efficacy statements **(B)** plotted as a function of contemporaneous goal status. The left panel **(A)** specifically displays retrospective/contemporaneous differences scored such that lower numbers indicate a decline from the earlier to the later period (Analogous difference scores were not computed for verbal responses because the retrospective sentiment scores were extremely negatively skewed.).

Figure 2 displays self-efficacy beliefs among participants with each of the three goal pursuit statuses; specifically, it displays both (a) changes in quantitative self-efficacy ratings (retrospective versus contemporaneous) and (b) qualitative analysis, namely, the sentiment analysis of contemporaneous self-efficacy verbalizations. The groups differed significantly on both the quantitative [*F*(2, 39.6) = 11.0, *p* < 0.001] and the qualitative indices [*F*(2, 35.2) = 5.32, *p* < 0.01]. However, that pattern of differences varied from one to another. On the quantitative self-ratings, particularly large declines in self-efficacy were observed among two subgroups: those who had abandoned and who were undecided about their project pursuit. However, in the sentiment analysis, the most negative scores were displayed by the undecided participants. Some undecided participants expressed multiple negative thoughts when verbalizing beliefs about their ability to pursue their goal, for example, “I’m not sure it’s what I want anymore because I am having a complete rethink about what is important in life. I felt in February that I had slightly overcommitted for this next year, and this has made me reconsider the extent to which I want to keep working. Maybe I have actually retired.”

A second, complementary way of examining relations between self-efficacy beliefs and goal pursuit is to explore goal pursuit status as a function of participants’ responses to the multiple-choice self-efficacy survey items “Can you still do this?” and “Are you pursuing the task now?” Results revealed a conflict: more than double the people who answered positively to the question “Do you think you can still do this, now that current events have unfolded?” (*n* = 51) confirmed that they were still pursuing their goal (*n* = 118). Table 2 relates these contemporaneous self-efficacy judgments and goal pursuit responses. As shown, almost all participants who retained confidence in their ability to achieve their goal reported that they were still pursuing it. Yet, interestingly, a great many participants who expressed a lack of confidence had *not* abandoned their goal. Factors that include, yet go beyond, reflections on self-efficacy may have influenced the continued pursuit of personal projects.

**TABLE 2:** Contemporaneous self-efficacy and goal pursuit.

	**Still pursuing goal**			
**Can you still do this [goal] now?**	**Yes**	**Unsure**	**No**	**Total**
Yes	46	1	4	51
Unsure	28	11	14	53
No	44	7	6	57
Total	118	19	24	161

Responses from those who reported with certainty either abandonment (no) or continuation (yes) to pursuing their goals, where the goals were not travel-related, and all that had similarly low self-efficacy scores (≤40) are presented in Tables 3, 4. Avoiding travel-related goals, which were essentially banned during this lockdown period, allowed for examination of goals that could have the possibility of strategic coping in relation to goal pursuit. The cutoff of 40 for self-efficacy scores encompassed all of those abandoning goals with low self-efficacy; those continuing to pursue their goals with similarly low self-efficacy are also represented in the table. Those who abandoned their goals and reported zero self-efficacy verbally close down the possibility of pursuing a goal. This language demonstrated a lack of strategic thinking, and in line with the self-efficacy theory (Zimmerman et al., 2017), these people quit when faced with the challenge of lockdown. In essence, they were unable or unwilling to engage in strategic behavior to find another way. However, those who confirmed they continued actively pursuing their goal, yet reported zero self-efficacy, often phrased their reasons with the future tense using words like “going to.” They acknowledged the *possibility* of pursuit exists, even if they were uncertain how this would occur.

**TABLE 3:** Non-travel goals abandoned and pursued for those with self-efficacy scores of 0.

**Category**	**Score**	**Reason abandoned**	**Category**	**Score**	**Reason pursuing**
E	0	It’s impossible. However, now that I think about it, I am still fulfilling professional obligations that I would have done there, just without the travel, time commitment, expense, and overwhelm of a large conference.	PFC	0	I’m pursuing ways to find other opportunities for alternative musical/professional work. This has moved exclusively to an online format. The overwhelming change to life has meant that each one of us has a glimpse of how life can fundamentally change in an instant - that new uncertainty also means that deferring plans (i.e., for 6 months or 1 year) also carries with it immense uncertainty that was never present in my mind before. It means that now I’m going to try to find ways to make things work in a different way as well.
E	0	General consensus is that predicted grades will be accepted alongside evidence. As school is closed it is not possible to complete work.	PFC	0	I am planning online sessions with different groups of stakeholders during the period I have scheduled to be there
P	0	Time is precious and even if I have to suffer financial losses, I am going to spend it making things that matter to me and finding ways to share them. I will find ways to make money that require less of my soul.	H	0	Postponed to 2021. Once the COVID-19 situation is more clear
P	0	No - because this goal was very specific – I’m not dropping the overall aim of increasing cycling mileage and speed again - just need to focus on what I can currently do - and pick specific event/goal once circumstances change.	H	0	Not actively pursuing as everything on hold. Except for the plans for the kitchen. We’re sitting and still planning that on paper.
U	0	The level of uncertainty in risk with regard to gathering people in the same place is simply too high to move forward.	H	0	If/when things return to a sense of normality, I will resume contact with the organizations and community groups I was working with and return to my project. However, how long this will take, if it is achievable at all, is a different kettle of fish.
			H	0	Delaying the event until after the lockdown
			NC	0	Music is the happiness in my life. So I want to make music.

*Columns detail the categories for abandonment/method of pursuit (categories for abandonment reason: E, external; P, priorities; U, uncertainty; categories for pursuit explanation: H, holding; NC, no change; PFC, problem-focused coping), numerical self-efficacy score, and participant free-text reason for abandoning/pursuing their goal.*

**TABLE 4:** Non-travel goals abandoned and pursued for those with self-efficacy scores of 1–40.

**Category**	**Score**	**Reason abandoned**	**Category**	**Score**	**Reason pursued**
P	1	****If there is no job to pursue, why chase it. Turn energies elsewhere.	O	5	Again, need money or I will starve to death.
E	10	The specific goal is not practical.	PFC	8	Still checking websites and employment agency web sites
E	20	Well I will try to do the French, however, Choir tour and the concert are not going to happen.	O	10	“Yes” as in “I think about it every day but I’m still procrastinating.” There’s a lot of new roadblocks now. I don’t know exactly what but I’m scared to find out.
P	25	No - These goals are now almost bottom of my priority, staying healthy, keeping my flat and re-adapting my teaching business to being completely virtual are my main goals	PFC	10	Still calling friends
E	30	It’s not physically possible due to the lock down and am reluctant to spend savings until we have a better idea of my partners ability to stay in his job/find a new one. I am unable to work.	O	11	I am still planning on going back to school, I just don’t feel that I can make any kind of financial commitment to it while I am so uncertain about my employment, my schedule, my health, medical bills, etc.
P	40	I love my job, and I wouldn’t want the fact that I wish to pursue my goals now have a major impact on my colleagues. It is a matter of timing, and once the situation we find ourselves in currently has died down and hopefully we return to normal, it will be then that I re-evaluate the situation.	EFC	15	I am still writing my book and using this time to think about priorities and redefine orientations and identities. The crisis situation is unexpected but gives me a lot to think about on a broader scale. Can we as a society step out of the crazy market system we have allowed to become total, infesting the very core of our being. The air hasn’t been so clear in as long as I can remember.
			EFC	20	I need to get in the right mindset and fit it in will other tasks that I am undertaking.
			NC	20	Nothing has changed I am just waiting to see what happens after the current situation and what state the economy and housing market is in.
			PFC	30	I will continue to work toward my assessments, even though they are not for the best part of a year. I will try and arrange extra support with my teachers to ensure I am not lacking in quality where I will no longer have that contact time after this semester. I will be asking my Academic Advisor for some kind of personal recommendation so that I am able to get a job despite not graduating this year. I do not wish to jeopardize my overall university grade by doing the alternative assessments at such short notice.
			O	30	At this current point I do not know yet.
			PFC	32	I’m still applying, though for different jobs now. I’ve applied for several “key worker’ jobs like Tesco and farm roles. I’ve set up as an online tutor. I’ve signed up as a volunteer. No luck though

*Columns detail the categories for abandonment/method of pursuit (categories for abandonment reason: E, external; P, priorities; categories for pursuit explanation: EFC, emotion-focused coping; NC, no change; O, other; PFC, problem-focused coping), numerical self-efficacy score, and participant free-text reason for abandoning/pursuing their goal.*

Those who abandoned their goals yet had some self-efficacy still do not allow for the possibility of achieving the goal despite quantifying their self-efficacy beliefs. There is no use of active verbs, and they frequently use forms of “no” or “not” (e.g., “not possible”), whereas those pursuing goals with a very low self-efficacy score convey a sense of enduring, continuity, and even urgency by using “still” and “need” alongside active verbs. Those actively pursuing goals, even with low self-efficacy scores, demonstrate a noticeable sense of possibility that embraces now and the future.

## Discussion

Our results provide a unique “snapshot” of how the COVID-19 crisis disrupted individuals’ pursuits of personal projects in the early period of the lockdown. Self-efficacy ratings for goal pursuit plummeted. Analysis of verbal self-efficacy reports mirrored the numerical results, showing significant differences between pre-COVID-19 and current self-efficacy beliefs for achieving valued projects. Almost all participants still cared about their goal, yet more than a quarter of the sample either had abandoned it or reported uncertainty about further goal pursuit. Given that sustained pursuit of projects enhances well-being (Little, 1993, 2007), our results highlight a potential psychological toll of the biological pandemic.

The results also, more positively, reveal ways in which people coped successfully with the constraints of the lockdown. Many participants were still working toward their goal, and among these, many reported creative problem-focused and emotion-focused coping strategies that sustained goal pursuit. Our qualitative methods yielded a “library” of participant-provided pandemic-related coping strategies.^3^ In future work, this library of strategies could be provided to others as one element of an intervention to enhance citizens’ well-being in the face of major social disruption (cf. Skoufias, 2003).

Although diverse in many ways, our sample was economically privileged from a global perspective. Many pursued professional and leisure projects inaccessible to lower-income persons. An implication is that our data reveal beliefs about goals people considered meaningful, even aspirational, as opposed to strictly being need-based. Also, at this early stage in the pandemic, reported strategies for understanding and approaching goals did not yet reflect crisis-type coping behavior (Ben-Zur and Zeidner, 1995). Instead, we saw the amplification of perceived limitations to achievement, and the interaction between self-efficacy beliefs and action becomes at least convoluted, and sometimes conflicted.

The initial lockdown phase of this crisis presented unexpected, tangible obstacles for people and challenged the stability of their beliefs toward goals. The imposed restrictions upset the predictable fabric of everyday engagement, and some people were not in an obvious position to enact their reported pre-COVID-19 high self-efficacy and good intentions toward their goals (Gollwitzer and Sheeran, 2006). Overwhelmingly, people still cared about these tasks, but many did not have an available repertoire of appropriate strategies. The pandemic conditions highlighted the need for adaptability to maintain and accomplish goals, and reliance on existing everyday routines may be inefficient and simply impractical under these uncertain conditions.

Creative engagement is a vehicle through which strategic development can flourish, and existing within the established confines of habit neither fosters creativity nor is an option for successfully navigating current challenges. Actively seeking to develop strategic approaches through new learning, developing the “practice of practice” (Antonacopoulou, 2006, p. 5), and the creation and adoption of disruptive innovation take vision and time (Yu and Hang, 2010). It is therefore not surprising that some people, unable to see a direct way forward, effectively put their goals on hold or abandoned tasks completely. In principle, those who abandoned their goals could adopt similar emotional and physical coping strategies used by others. Developing new strategies and perspectives is an enduring challenge for all.

The assessment of self-efficacy in this research, gathering both the quantitative (numeric) and qualitative (dialogic free text) responses, is genuinely novel in the field of self-efficacy research. Our results demonstrated that both methods produced comparable, statistically significant results, and this is a pioneering contribution to the approach of self-efficacy measurement in psychological research. The present research extended the standard questionnaire-based approach to self-efficacy research, with the novel methodological approach of including free-text descriptions of self-efficacy beliefs alongside traditional scalar measures. Anchoring these text responses to both the numerical score and sentient analyses demonstrated that these free descriptions also produced significant results. This initial step to innovate methods opens the door for further research to explore the syntax of self-efficacy. Future research should aim to understand self-efficacy beliefs in terms of verbal expression, internal thought representation, and the expressed interrelationships between declared externalizations of self-efficacy to enacted beliefs (through tasks) to deepen the understanding of belief and achievement.

## Data Availability Statement

The datasets presented in this study can be found in online repositories. The names of the repository/repositories and accession number(s) can be found here: Open Science Framework https://osf.io/64y2p/.

## Ethics Statement

The studies involving human participants were reviewed and approved by University of Chichester Research Ethics Committee. The patients/participants provided their written informed consent to participate in this study.

## Author Contributions

LR: contributes to the concept, design, definition of intellectual content, data analysis, manuscript preparation, and manuscript editing. DC: contributes to the literature search, definition of intellectual content, manuscript preparation, and manuscript editing. BS: contributes to the design, data analysis, and manuscript editing. All authors contributed to the article and approved the submitted version.

## Conflict of Interest

The authors declare that the research was conducted in the absence of any commercial or financial relationships that could be construed as a potential conflict of interest.

## Footnotes

1 https://www.qualtrics.com/uk/

2 https://aws.amazon.com/comprehend/

3 See data file: https://osf.io/64y2p/
